# Spatial orientation of cross-sectional images of coronary arteries: point of view in intracoronary imaging

**DOI:** 10.1186/1476-7120-10-12

**Published:** 2012-03-22

**Authors:** Troels Thim, Erling Falk

**Affiliations:** 1 The Atherosclerosis Research Unit, Department of Cardiology, Aarhus University Hospital (Skejby) and Institute of Clinical Medicine, Aarhus University, Aarhus, Denmark; 2 Department of Cardiology, Aarhus University Hospital (Skejby), Brendstrupgaardsvej 100, DK-8200 Aarhus, Denmark

**Keywords:** Intracoronary imaging, Intravascular ultrasound, Optical coherence tomography, Tissue characterization

## Abstract

**Background:**

In studies where cross-sectional images of coronary arteries obtained with different imaging modalities are compared, the importance of correct co-localization and matching of images along the coronary artery longitudinal axis is obvious. However, it appears neglected that correct spatial orientation of the cross-sectional plane may not be obtainable just by rotating the images to ensure co-localization of identifiable landmarks such as sidebranches. A cross-section has two sides, one facing proximally and the other distally, and pairs of images reconstructed corresponding to these opposite points of view are mirror images of each other and not superimposable. This may be difficult if not impossible to recognize and unrecognized it will give rise to flawed results in the development and validation of imaging technologies aimed at plaque characterization (tissue mapping). We determined the imagined point of view for three commercially available intracoronary imaging systems used by invasive cardiologists and illustrate its importance in imaging modality validation.

**Methods and Results:**

We made an asymmetric phantom and investigated it with two different intravascular ultrasound (IVUS) systems and one optical coherence tomography (OCT) system. The asymmetry of the phantom allowed determination of the spatial orientation of the cross-sectional images. On all tested systems, an observer should imagine herself/himself standing proximal to the cross-section when looking at the intravascular images.

**Conclusions:**

The tested intracoronary imaging modalities displayed cross-sectional images with a spatial orientation corresponding to a proximal point of view. Knowledge of the spatial orientation is mandatory when comparing and validating different imaging modalities aimed at plaque characterization.

## Background

Many intravascular ultrasound (IVUS) and optical coherence tomography (OCT) cross-sectional images of coronary arteries are published in biomedical journals. Sometimes these images are presented together with images from microscopic examination of the same arterial positions illustrating how the imaging modality correctly displays tissue characteristics [1,2].

When comparing cross-sectional images obtained with different techniques, such as intravascular imaging and microscopy, efforts must be made to assure that the images are from exactly the same arterial position along the longitudinal axis of the coronary artery, and this is often devoted a lot of attention.

Also, when comparing cross-sectional images, rotational adjustment is usually required to obtain correct co-localization of identifiable landmark structures such as sidebranches. Rotation of a cross-sectional image does not change area measurements or intraplaque location of a certain component of interest. Therefore rotation does not affect interpretation.

However, in addition to longitudinal position and rotation, cross-sectional images also possess a spatial orientation of the cross-sectional plane. This is only rarely commented on but cannot be taken as lightly as rotation [3,4]. Changing spatial orientation, and thereby our imagined point of view, changes intraplaque location of plaque components in the cross-sectional plane. Thereby spatial orientation has implications in studies focusing on the identification and localization of plaque components (tissue mapping).

We determined the spatial orientation of cross-sectional images displayed on two different IVUS systems and one OCT system and exemplify implications.

## Methods

### Spatial orientation of microscopic sections

**Figure 1:**
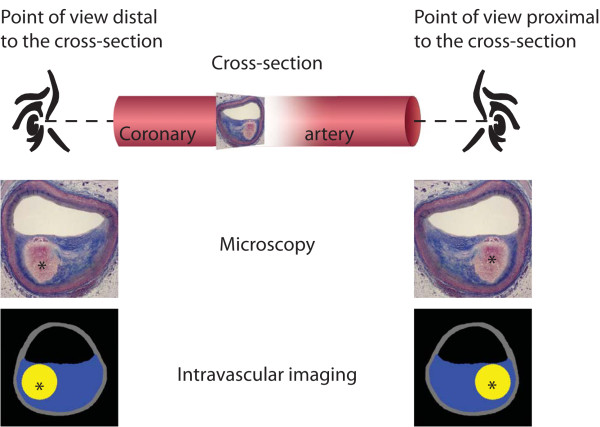
**Cross-section of a coronary artery containing a thin-cap fibroatheroma (TCFA)**. Images from microscopy are shown at the top, images obtained with an intracoronary imaging technology at the bottom (necrotic core displayed in yellow (asterisk), fibrous tissue in blue). The observer can imagine herself/himself looking at the cross-sectional image standing either proximal or distal to the cross-section. From these two opposite imagined points of view the observer sees mirror images, i.e. the images have different spatial orientations and are not superimposable. Although a TCFA is seen from both points of view, only images viewed from the same point of view have similar intraplaque localization of plaque components. Comparing intraplaque location on images only makes sense if seen from the same point of view. Microscopy: trichrome stain (collagen blue, necrotic core colorless).

When a section is cut for microscopic examination, the section may be laid down on the glass slide on one of two sides. Photomicrographs of a section lying on different sides will be mirror images of each other. So, although they are photomicrographs of the same section, i.e. similar position and rotation, the intraplaque localizations of plaque components will differ (Figure 1).

With strict adherence to procedures throughout the preparation of arterial sections for microscopic examination, the same known spatial orientation of sections can be obtained. Thereby, with proper attention to orientation in the preparation of tissue section for microscopy, one can always decide and know the imagined point of view, i.e., whether one should imagine looking at a coronary artery cross-section standing proximal (at the coronary ostium) or distal to the cross section (Figure 1).

### Spatial orientation of intravascular images

An intravascular imaging catheter looks from the arterial lumen and outward on the arterial wall. But the displayed images created with intravascular imaging represent cross-sections that have one of two possible spatial orientations, just like the microscopic section. Throughout the pull back, the observer should imagine herself/himself standing either proximal or distal to the cross-section when looking at the cross-sectional images (Figure 1). The spatial orientation and thereby the imagined point of view is decided by the reconstruction algorithm. When making comparisons of intravascular and microscopic images, one has to know the spatial orientation of the intravascular images in addition to the images from microscopy and make sure they are the same before any comparison is meaningful. To know the spatial orientation, one has to either know the reconstruction algorithm or determine the spatial orientation experimentally.

### Revealing point of view

**Figure 2:**
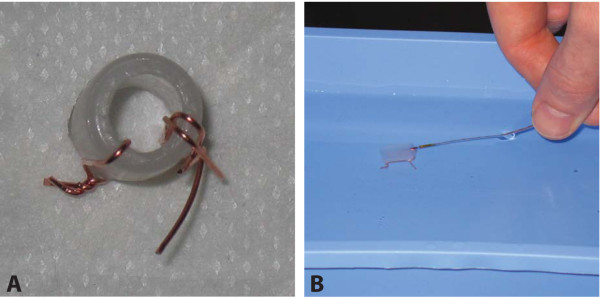
**Methods**. A. Asymmetric phantom seen from the imagined proximal end. One wire at 7-8 o'clock and two wires at 4-5 o'clock creates the asymmetry. B. With the phantom submerged in water, both intravascular ultrasound (IVUS) catheters were advanced into the phantom through the imagined proximal end.

To determine the spatial orientation experimentally, we made an asymmetric phantom using a short piece of tubing with two wire points not directly across from each other on the cross-section of the tube. One wire alone constituted one wirepoint and two wires close together constituted the other. We then decided which end was to be considered the proximal and investigated the tube with two different IVUS systems and one OCT system inserting the IVUS and OCT catheters from the imagined proximal end (Figure 2).

The IVUS systems were Volcano In-Vision Gold system with an Eagle Eye Gold catheter and Boston Scientific iLab system with an Atlantis SR Pro catheter. The IVUS investigations were performed with the phantom submerged in water. Manual pull back recordings were used. The OCT system was Light Lab C7XR with a Lightlab C7 Dragonfly Imaging Catheter.

## Results and discussion

### The spatial orientation (point of view)

**Figure 3:**
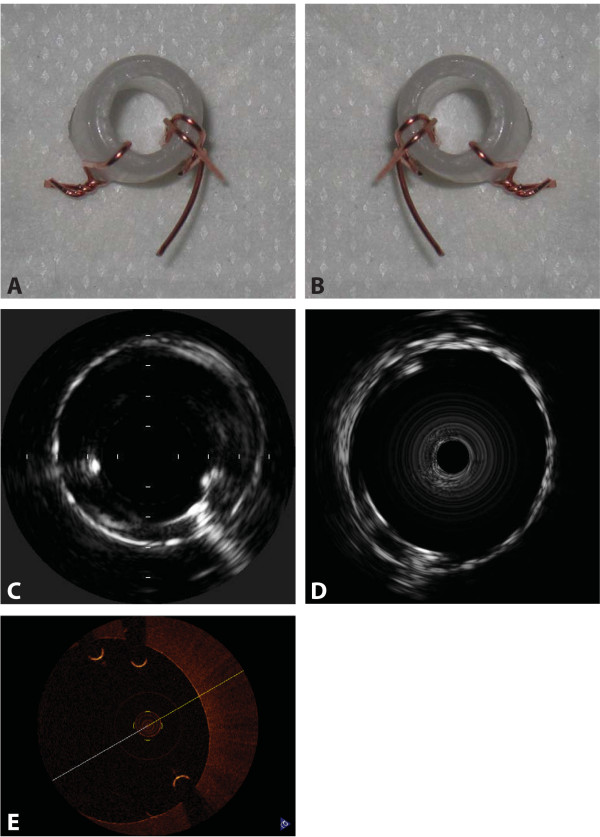
**Results**. A. Asymmetric phantom seen from the proximal point of view. B. Mirror image of A illustrating the distal point of view. C. Volcano IVUS showing the one wire at 8 o'clock and the two wires at 4 o'clock. D. Boston Scientific IVUS showing the one wire at 11 o'clock and the two wires at 7 o'clock. D. Light Lab OCT showing the one wire at 4 o'clock and the two wires at 12 o'clock. Rotation will make the images in C, D, and E superimposable on A but not on B (the mirror image), i.e., the cross-sectionel images displayed by the three intravascular imaging methods are viewed from the proximal point of view.

On the IVUS and OCT images, all three wires were clearly visible making the two asymmetrically placed wire points clearly distinguishable. Owing to the asymmety of the phantom, it was evident that the intravascular images were displayed to the observer, so that the observer should imagine herself/himself looking at the cross-sectional images standing proximal to the displayed cross-sectional image of the artery (Figure 3).

In studies comparing cross sectional images from these imaging systems to images from microscopy, investigators should then ensure that their cross sectional images from microscopy display the proximal view (same spatial orientation). Otherwise, comparison will not be meaningful.

### When does knowledge about spatial orientation not make a difference?

Clinically, treatments, such as angioplasty or stenting, are applied equally to the entire arterial circumference. Therefore, for making the clinical decision whether to apply a treatment or not, the spatial orientation of the cross-sectional image does not make a difference.

In clinical research, area measurements on intravascular images are used as study end points [5]. Measurements on areas, e.g. of a plaque or necrotic core from reconstructed images, yield the same results regardless of the spatial orientation and therefore the thin-cap fibroatheroma (TCFA)[6,7] can be correctly identified from both points of view (Figure 1).

However, meaningful use of an intravascular imaging modality, both clinically and in clinical research, provides that the intravascular imaging modality has been properly validated for identification and quantification of the plaque components that are of interest clinically and in clinical research. I.e., the use of these imaging modalities clinically and in clinical research is dependent upon reliable validation of the imaging systems. It is in this validation that spatial orientation is important.

### When does knowledge about spatial orientation make a difference?

**Figure 4:**
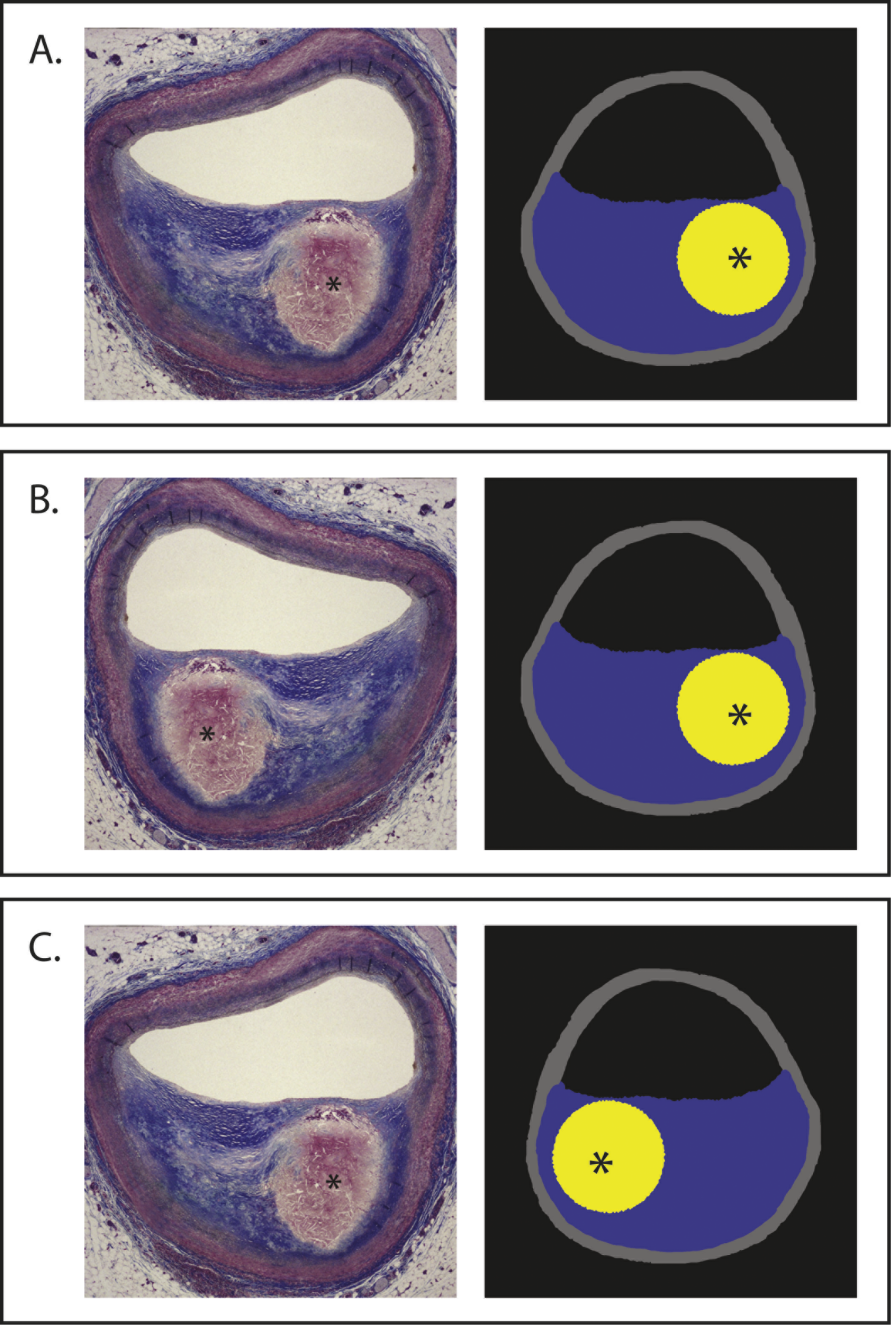
**Evaluation of tissue characterization software**. Cross-sectional images from software displaying the necrotic core (asterix) in yellow and fibrous tissue in blue are compared to images from miscroscopy. In A, the software correctly detects the size and intraplaque location of necrotic core which is not the case in B and C. However, these conclusions are only valid if we know that the spatial orientation of the compared cross-sectional images is the same.

For validation of imaging modalities focusing on the identification and quantification of plaque components, intraplaque localization is used and the spatial orientation of the cross-sectional images becomes critical (Figure 4). When the images from intravascular imaging and microscopy have the same spatial orientation, comparison makes sense. But if the images from intravascular imaging and microscopy have different spatial orientations, the compared images are not superimposable and direct comparison makes no sense. The spatial orientation can and must be known before any comparison of the images because knowledge about spatial orientation is crucial for correct interpretation of cross-sectional images and validation of technologies creating such images (Figure 4).

These considerations apply to the comparison of asymmetric cross-sectional images in general, irrespective of imaging modalities. In this study, other imaging modalities such as computed tomography, magnetic resonance imaging, near infrared spectroscopy etc., were not investigated; however the considerations about spatial orientation also apply whenever images from these modalities are compared to images from other imaging modalities, e.g. microscopy.

### Intravascular imaging catheter rotation

Intravascular imaging catheters can rotate along their longitudinal axis during pull back. Therefore, spatial orientation cannot be determined based on localization of two structures present on different images from the same pull back. Neither can spatial orientation be determined based on the circumferential localization of a single landmark, such as a sidebranch, as catheter rotation randomly determines the localization of this landmark. Determining spatial orientation experimentally requires asymmetry in one image.

## Conclusions

The tested intracoronary imaging modalities displayed cross-sectional images with a spatial orientation corresponding to an imagined point of view proximal to the cross-section. Knowledge of the point of view is mandatory when comparing and validating different imaging modalities. These considerations have implications for the development and evaluation of imaging technologies. Spatial orientation of images might appropriately be disclosed by manufacturers and discussed in consensus papers on intravascular imaging [8,9].

## Abbreviations

IVUS: Intravascular ultrasound; OCT: Optical coherence tomography; TCFA: Thin-cap fibroatheroma.

## Competing interests

The authors declare that they have no competing interests.

## Authors' contributions

TT: conception and design, acquisition of data, and analysis and interpretation of data, drafting of the manuscript. EF: analysis and interpretation of data, revision of the manuscript. Both authors read and approved the final manuscript.
